# How to build the climate resilience and environmental sustainability of your facility and services

**DOI:** 10.4102/phcfm.v18i1.5445

**Published:** 2026-05-25

**Authors:** Robert J. Mash, Patricia N. Schwerdtle

**Affiliations:** 1 Division of Family Medicine and Primary Care, Faculty of Medicine and Health Sciences, Stellenbosch University, Cape Town, South Africa; 2 Heidelberg Institute of Global Health, Faculty of Medicine, Heidelberg University, Heidelberg, Germany; 3 Climate Action Accelerator, Geneva, Switzerland

**Keywords:** climate resilience, environmental sustainability, primary care, district hospitals, carbon footprint

## Abstract

African district-level health facilities and services face many interconnected challenges including infectious disease outbreaks, conflict and insecurity, fiscal austerity, population displacement and climate-related hazards such as heat, flooding and drought. Emergency preparedness and disaster risk reduction plans have often not included the challenges from climate hazards. In this article, we outline six practical steps that a facility can take to assess its vulnerabilities, capacities and climate-related risks, and develop a plan to improve climate resilience and environmental sustainability: (1) a desktop review of the facility and services; (2) contextualisation and planning; (3) an audit of the facility; (4) a focus group discussion; (5) integration and analysis; (6) creation of an action plan. Environmental sustainability needs to be considered simultaneously with climate resilience as the key domains are the same (workforce; energy; water, waste and sanitation; infrastructure and equipment). In addition, the article outlines how to perform a carbon footprint and integrate the findings into one action plan for the facility. The article provides a practical roadmap for evaluating and improving the climate resilience and environmental sustainability of primary care or primary hospital facilities, and readers can access the referenced resources if they need additional details and support.

## Introduction

African district-level health facilities and services face many challenges including infectious disease outbreaks, conflict and insecurity, fiscal austerity, population displacement and climate-related hazards such as heat, flooding and drought.^1^ These hazards rarely occur in isolation, but can be compound (multiple hazards occurring simultaneously), cascading (one hazard triggering or amplifying others) or interacting with existing social, economic and health system vulnerabilities to increase overall risk.^2^ While emergency preparedness and disaster risk reduction plans often address acute shocks, they have not always adequately accounted for climate change as a current and intensifying risk multiplier – one that reshapes the frequency, severity and interaction of hazards already affecting primary care delivery.

Climate change is altering the burden and distribution of disease, changing the morbidity patterns seen in clinical practice.^3^ Climate hazards, such as heat waves, drought, wildfires, storms, cyclones and flooding, impact communities directly and place growing strain on the primary care facilities and primary hospitals that serve them. Facilities may be damaged or even destroyed by climate hazards, and healthcare workers may struggle to get to work or be stranded at the health facility. Essential services such as energy, water and sanitation may be disrupted, as well as the supply chain and communications. Facilities may also be required to operate under extreme temperatures that impact patients, healthcare workers and pharmaceutical supplies. Local populations may be displaced or suffer from climate-sensitive disease outbreaks, such as gastroenteritis or cholera. Healthcare workers are therefore often required to respond to acute climate-related emergencies while maintaining routine essential services, increasing operational pressure on already constrained health systems.

In their latest framework for health systems from a primary healthcare lens, the World Health Organization (WHO) identify three key components in service delivery.^4^ The model of care looks at what services are provided and how they are organised, key systems address quality improvement and patient safety, while the third area looks at the resilience of facilities and services. Resilience has usually been considered in relationship to infectious disease outbreaks and pandemics, but given the impact of climate change, climate resilienNote: The manuscript forms part of the themed collection titled ‘Continuing professional development for planetary health’, guest edited by Prof. Robert Mash.ce is also important.

Ironically, health systems also contribute to climate change through their own carbon emissions. Globally, the health sector contributes about 5% of emissions.^5^ While Africa’s absolute contribution is relatively small, the intensity of carbon per dollar of health spending is often high,^6^ reflecting inefficiencies in energy use, infrastructure and supply chains. If unaddressed, this creates a risk that future health system expansion will lock countries into high-cost, high-waste, fossil fuel-dependent models of care. For African health systems, this is not simply a question of environmental responsibility – it is a smarter development choice. Replicating the historical pathways of high-income countries has produced systems that are expensive to run, vulnerable to energy and supply shocks, and poorly adapted to climate stress. By contrast, consciously pursuing a low-carbon development pathway offers an opportunity to build health systems that are more affordable, more efficient and more resilient.

As countries work to strengthen the climate resilience of primary care facilities and services, environmental sustainability must be considered alongside resilience, not as an add-on but as a core design principle for future-ready health systems. In this article, we describe an approach to evaluating the climate resilience of our facilities and services with a focus on primary care and primary hospitals. The approach should culminate in an action plan. At the same time, we outline an approach to evaluating the carbon footprint and looking at ways of improving environmental sustainability. Family physicians often play a key role in leadership and need to contribute or even lead such initiatives.

## Evaluating climate resilience

Climate resilience has been defined as the ability to prepare for, respond to and recover from climate-related shocks and stressors.^7^ Recovery should ensure the facility returns to at least its baseline level of performance. A health system may learn from the experience and recover in a way that strengthens its performance and increases its resilience for future hazards. We describe a six-step process developed by the Climate Action Accelerator initiative and readers can refer to their online manual for more detail.^8^ The approach has been developed and field tested in low-resource and challenging situations such as a hospital in Chad and refugee camps in Bangladesh as well as primary care in South Africa:^8,9^

- A desktop review of the facility and services.
- Contextualisation and planning.
- An audit of the facility.
- A focus group discussion.
- Integration and analysis of the findings.
- Creation of an action plan.

This process can be followed for a single facility or several facilities in a subdistrict.

**FIGURE 1:**
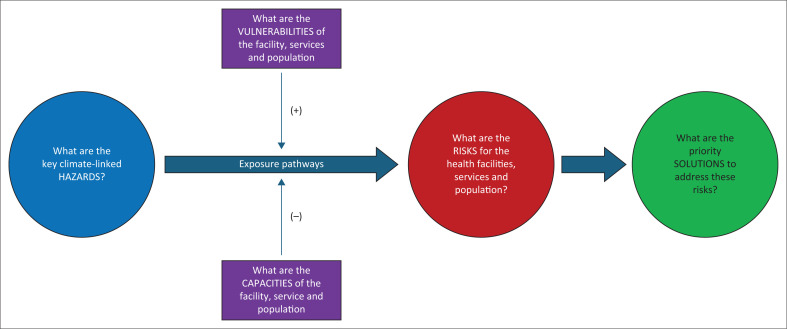
Conceptual framework.

The underlying conceptual framework is shown in Figure 1. The process should identify the likely current and future climate hazards in the local area. The vulnerabilities and capacities of the facility and services to succumb to or withstand these hazards should be considered. Vulnerabilities are the factors that make a facility or service more likely to be harmed by a hazard (such as weak infrastructure, staff shortages or unreliable power or water supply). Capacities are the strengths, resources and systems that enable the facility to anticipate, withstand, respond to and recover from these hazards. The actual risks to the facility and services will depend on the climate hazards, their exposure to these hazards and how the impact of these hazards are modulated by the vulnerabilities and capacities. Once the risks are identified, then potential solutions can be listed to reduce these risks. A final action plan will prioritise the most feasible and effective solutions. You may need a small team to assist you in the process. Such a team should include the relevant managers and leadership.

### A desktop review of the facility and services

Before gathering new information, you should consider what you already know. However, information on climate change in your local area may need to be sought from local government, national departments of the environment or even international bodies. This should help to confirm the most likely climate hazards in your area. Consider also the characteristics of the community served, burden of disease in your community and morbidity profile. Think how this may be impacted by the climate hazards. Gather information also on the workforce, workload and infrastructure. This information will help identify the known vulnerabilities and capacities. Summarising the key points in a short document will help consolidate the information and inform the next steps.

### Contextualisation and audit of the facility

There are several key areas to consider in the audit (see Table 1 for examples):

- The workforce.
- Energy.
- Water, waste and sanitation.
- Infrastructure and equipment.
- Emergency preparedness.
- Service delivery.

**TABLE 1:** Key issues to consider in the audit.

Domain	Examples of key issues to audit
Workforce	Number and type of health care workers in the facility and local community, number of vacant posts Climate smart work practices, e.g. plan for managing extreme temperatures Resilience of the workforce during sudden peaks in demand such as infectious outbreaks or mass casualties, e.g. numbers available, adjusted hours, psychological and practical support Training on climate health, e.g. health effects of climate change, climate resilience, environmental sustainability
	Energy	Energy supply, e.g. reliability, energy storage, alternative or renewable energy sources Energy management, e.g. what is supplied by alternative systems, effectiveness of maintenance and repair, type of air conditioning, use of geysers or boilers, initiatives for energy efficiency
		Water	Water supply and reliability, water storage, alternative or emergency water supplies, effectiveness of maintenance and repair
	Waste	Segregation of general, anatomical and non-anatomical hazardous waste, use of incineration, landfill, composting and recycling
Sanitation	Toilet functioning and disposal of sewage during climate events, cleaning of the facility
Infrastructure	Number of buildings and rooms, overall state of the structure, stability of roof, hazardous materials (e.g. asbestos), colour of roof, insulation, hazardous trees, flood protection, minimum and maximum temperatures in facility and pharmacy, effectiveness of maintenance and repair
Equipment	Type of oxygen supply and reliability, biomedical equipment, effectiveness of maintenance and repair
Emergency preparedness	Emergency preparedness plan, e.g. is it available and updated, are staff trained, does it include medical supplies, laboratory services, communication systems, stock management, consideration of climate hazards, continuity of care if facility not accessible or usable, any early warning systems Community and stakeholder engagement, e.g. is there a local disaster management committee, do staff participate, is there community engagement Health information, e.g. any early warning systems, surveillance of climate-sensitive conditions, response to trends in routine data
	Service delivery	Climate smart processes and protocols, e.g. infection prevention and control, changes to patient flow, emergency medical services Outreach services, e.g. community engagement and health promotion on climate-related issues

Practical tools are available from the Climate Action Accelerator,^8^ and the audit tool should be adapted to the local context. Adaptation may also consider the comprehensive list of issues in the WHO guidance.^7^ It should be possible to complete the audit for a facility in a 60-min interview with the manager and a brief walk through to directly observe some of the key issues. Photographs of key infrastructure and issues may supplement the audit. The audit can be documented in an Excel spreadsheet as numerical or categorical answers. Additional explanatory notes are helpful.

### A scenario-based tabletop focus group discussion

**FIGURE 2:**
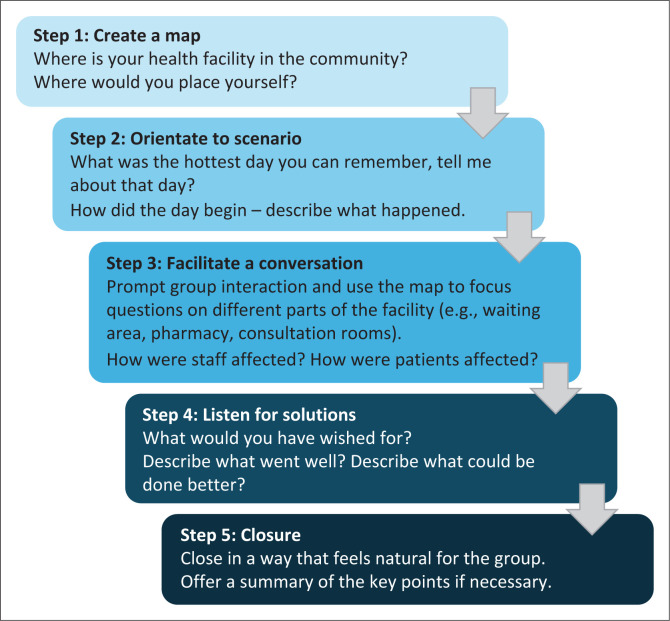
Focus group discussion process.

The audit information can be supplemented by discussion and feedback from the staff. Gather a group of 8–10 people from each facility. Ideally this should include clinical staff, support staff, managers and some community-based healthcare workers. One person should facilitate the discussion, while a second person listens and makes notes. You may also want to record the conversation to help with the summary later. The discussion can follow a five-step process as shown in Figure 2. The focus of the discussion is on a previously experienced climate event (e.g. extreme heat, flooding) and an exploration of how the staff experienced it, how they coped and what would help in future events.

The observer should listen for key points in the discussion that signify areas of weakness or vulnerability, areas of strength or capacities and potential adaptations or solutions. After the discussion, the observer should look at their notes, listen to the recording and construct a 2–3-page summary of the key vulnerabilities, capacities, risks and solutions that came up in the discussion.

### Integration and analysis of the findings

The results of the audit(s) and the focus group discussion(s) should be combined in an Excel spreadsheet or matrix (Table 2). The first column identifies the key climate hazards. For each hazard, the next columns list the identified vulnerabilities and capacities and resultant risks. Once the risks have been identified, then a final column can insert potential solutions. Solutions may be implied or derived from the audit and discussion but may also be identified from the literature and experts. Solutions may also be critiqued by technical experts if they are available.

**TABLE 2:** Example of the analysis matrix.

Climate hazards	Vulnerabilities	Capabilities	Risks	Potential solutions
Extreme heat and drought	Roofs do not reflect heat	Several clinics have white tiles but not highly reflective	Increase in facility temperature and increased use of energy to cool	Paint roofs with highly reflective white paint.
	During loadshedding the alternate energy supply does not include air conditioners	All clinics have window-based air conditioning units	Inability to cool clinic during loadshedding or power cuts	Review policy for high temperatures. Increase efficiency of natural ventilation.
	Open window policy reduces effectiveness of air conditioning	-	High temperatures in clinic vs reduced ventilation for tuberculosis risk	Review policy for high temps. Increase efficiency of natural ventilation.
	Patients waiting outside in the heat may be vulnerable	Four clinics have sufficient shade areas for usual workload	Patients’ condition may worsen when waiting outside in extreme heat	Ensure that every clinic has sufficient shade for waiting outside.
	Farm and manual labourers working in extreme heat	Mobile clinics go to farms and community health worker teams cover communities	Manual and farm labourers at risk of heat related diseases, dehydration, heat exhaustion, stroke	Health promotion in farms (other workplaces) on action to take and modification of work patterns during extreme heat. Occupational health assessments.

### Creation of an action plan

The potential solutions can be extracted from the matrix and organised into six domains:

- Health workforce.
- Energy.
- Water, waste and sanitation.
- Infrastructure and equipment.
- Service delivery.
- Governance and financing.

The management and leadership of the facility together with the evaluation team should rank and prioritise the suggested solutions. Key considerations would be cost, feasibility, impact on climate resilience, other environmental benefits, responsibility of other stakeholders and alignment with existing priorities. Criteria can be selected according to the local context, and a simple scoring system for each criterion (e.g. low, moderate, high) can assist with prioritisation. The solutions in each category can be considered in turn. This should lead to a much smaller list of solutions that can be included in an action plan. Some may be immediately implementable in the short term, while others may need additional financing and planning with the department of health or even external funders.

## Evaluating the carbon footprint

The WHO consider climate resilience and environmental sustainability as two sides of the same coin as they both relate to the same set of key issues: the workforce; energy; water, waste, sanitation; infrastructure and equipment.^7^ These essential elements of the facility may be impacted by climate hazards, but also contribute to environmental impact and the carbon footprint. Solutions can improve both resilience and sustainability. Evaluating the carbon footprint may feel like a difficult and highly technical task. However, the organisation Health Care Without Harm (HCWH) have come up with a method and online tool that make the job relatively easy.^10^ The tool can also be downloaded as an Excel spreadsheet. The carbon footprint categorises the facility’s contribution to greenhouse gas emissions into three areas or scopes (Table 3).

**TABLE 3:** Categories of greenhouse gas emissions.

Scope 1	Scope 2	Scope 3
Direct emissions from the use of fossil fuels or greenhouse gases at the facility:	Indirect emissions from the generation of electricity, steam, heating or cooling:	Indirect emissions because of the activities at the facility:
• Stationary combustion, e.g. generator	• Purchased electricity	• Extra supply chain
• Mobile combustion, e.g. vehicles	• Purchased steam, heat or cooling	• Business travel
• Fugitive emissions, e.g. gas in fridges or air conditioning	-	• Electricity transmission and distribution losses
• Waste treated on site, e.g. incineration	-	• Inhalers
• Anesthetic gases	-	• Waste management off site
-	-	• Employee commuting
-	-	• Patient commuting

*Source:* Adapted from Health Care Without Harm. How to use climate impact checkup [homepage on the Internet]. 2023 [cited 2025 Oct 22]. Available from: https://greenhospitals.org/checkup

Data need to be collected for each of the key items in Table 4, and more detail is given in the Manual.^10^ Some items may not be relevant and can be excluded from the calculation, while the data for other items may be too difficult to obtain. Commuting may need to be estimated based on a short survey with staff and patients. The more items that can be included, the more accurate the final footprint, but if items are excluded, this should be stated as a limitation of the final footprint report.

**TABLE 4:** Data needed for a carbon footprint.

Item	Data needed
Stationary combustion	Total amount of fuel type consumed by generators
Mobile combustion	Total amount of fuel type consumed by vehicles
Fugitive emissions	Type of equipment, type of gas and quantity of gas leaked or refilled
Medicinal and anaesthetic gases	Total amount of different gas types delivered and discarded
Purchase of electricity	Total amount consumed in Kwh
Purchase of steam, heat or cooling	Quantity of steam or water
Business, staff and patient travel	Type transport, distance, frequency or number of trips
Inhalers	Types of inhalers, number dispensed and prescribed
Electricity transmission and distribution	Extrapolated from total amount consumed (no data needed)
Extra supply chain	Amount spent on different categories
Solid waste	Amount of waste to landfill
Composting	Amount of waste
Incineration	Amount of waste

*Source:* Adapted from Health Care Without Harm. How to use climate impact checkup [homepage on the Internet]. 2023 [cited 2025 Oct 22]. Available from: https://greenhospitals.org/checkup

**FIGURE 3:**
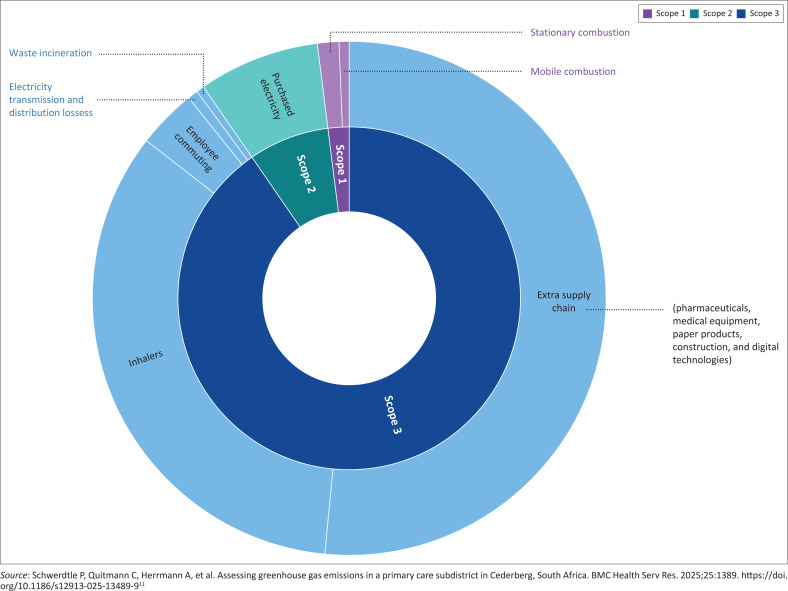
Example of a carbon footprint of primary care facilities in the Cederberg subdistrict, South Africa.

The HCWH Climate Impact Tool uses the data entered to calculate greenhouse gas emissions by applying emissions factors to each activity (such as fuel use, electricity consumption or travel). Where possible, these emissions factors are country-specific, reflecting local energy sources and conditions. If country-specific emissions factors are not available, global averages are used. The final carbon footprint can be presented as a total annual amount of carbon dioxide equivalent (eCO_2_), an amount of eCO_2_ per bed or per consultation and the proportions of the footprint allocated to different scopes (Figure 3). The analysis and reporting are automated by the online tool, reducing the technical burden for users. Importantly, the results are not intended to sit on a shelf. Healthcare Without Harm provides guidance and resources to help facilities identify priority areas for action and translate their carbon footprint into practical mitigation measures. These may include ‘quick wins’ – such as improving energy efficiency, reducing waste or changing procurement practices – as well as longer-term investments, such as transitioning to renewable energy, upgrading infrastructure or redesigning models of care. The footprint from the Cederberg (Figure 3) highlighted the potential for reducing the carbon footprint by changing the prescription of inhalers from metered dose to dry powdered inhalers. In this way, reducing the carbon footprint can directly inform the facility action plan, support environmental sustainability while also strengthening the resilience and efficiency of health services.

## Conclusion

Family physicians and facility-level healthcare teams are well placed to lead practical action on climate resilience and environmental sustainability within primary care facilities and primary hospitals. This article describes practical steps to assess climate risks, identify priorities and develop facility-level action plans. At the same time, facilities can strengthen these efforts by considering their carbon footprint and adopting low-carbon development pathways. Taking an integrated approach – one that addresses resilience and sustainability together – can help protect patients, support healthcare workers and build stronger, more resilient and future-ready health services.
